# Characteristics of overburden failure and evolution of fractures in fully mechanized top coal mining face

**DOI:** 10.1038/s41598-025-21281-2

**Published:** 2025-10-27

**Authors:** Xiaolei Wang, Fengzhe Liu

**Affiliations:** Department of Resources and Mechanical Engineering, Lyuliang University, Lvliang, China

**Keywords:** Mining engineering, Overburden failure, Microseismic monitoring system, Cracks evolution, Energy science and technology, Engineering, Solid Earth sciences

## Abstract

The characteristics of overlying strata failure and the evolution law of fractures during coal mining are of great significance for mine water prevention and gas control. To further study the overlying strata failure characteristics and fracture evolution in fully mechanized top-coal caving (FMTC) mining faces, a Shanxi coal mine was selected as the experimental site. Methods such as segmented water injection, borehole television imaging, and microseismic monitoring were employed to detect the failure height of the overlying strata. A digital analysis was conducted on the relationship between fracture dip angle, quantity, and width before and after mining, along with numerical simulations of fracture evolution during the mining process.The results show that the overlying strata failure height in the FMTC mining face ranges from 127.3 m to 132.2 m. Before mining, fracture development was relatively low, whereas after mining, the number of fractures significantly increased, indicating enhanced fracture development. Pre-mining fractures were primarily characterized by high angles and low widths. As the coal face advanced, the number of fractures rose linearly, with the newly formed fractures mainly exhibiting small angles and medium widths. During mining, fractures predominantly concentrated around the coal wall, and the density distribution curve of overlying strata fractures showed a “wave-like” pattern, with higher values at both ends and the middle.

## Introduction

 The characteristics of overlying strata failure and the formation and evolution of fractures during coal mining are critical issues of significant concern in mining engineering^1^. The overlying strata failure induced by coal mining often leads to geological disasters such as surface subsidence and fracture propagation, posing severe threats to miners’ safety and the sustainable development of mines. Research on this issue not only pertains to the efficient extraction and utilization of coal resources but also relates to ecological protection and the sustainability of safe mining practices^2^.

Analyzing the failure characteristics of overlying strata in coal mining enhances our understanding of the stress distribution and deformation patterns of rock layers during extraction, providing essential references for designing scientifically sound support measures^3^. Simultaneously, studying the evolution laws of fractures is crucial for predicting and preventing geological disasters. By gaining insights into the dynamic evolution of fractures, the adverse impacts of mining activities on the surrounding environment and personnel safety can be effectively mitigated^4^.

Exploring the failure characteristics of overlying strata and fracture evolution requires integrating knowledge from disciplines such as geomechanics and geotechnical engineering, along with modern technological methods for data acquisition and analysis. Only through systematic and in-depth research can we better predict and control geological disaster risks during coal mining, thereby ensuring safe, stable, and sustainable mine production^5^.

Numerous scholars worldwide have conducted extensive research on the failure characteristics of overlying strata. For instance, taking No. 2 Zhundong Coal Mine as the research object, the failure characteristics of its overlying strata were explored. By analyzing the strata pressure behavior characteristics and microseismic data, they studied the relationship between roof failure and strata pressure. The results show that this method can effectively evaluate the failure characteristics of overlying strata and provide important support for the safe mining of coal mines^6^.Focusing on the failure characteristics of overlying strata in deep strip goafs, a “point line surface volume” multi-parameter monitoring model was constructed by comprehensively applying technologies such as borehole television imaging, optical fiber sensing, cross-hole electrical CT, and microseismic monitoring. Practice has proved that this method has high stability and accuracy and can meet the needs of overlying strata deformation monitoring^7^. In-depth research was carried out on the overlying strata failure problem under repeated mining of shallow coal seams in a coal mine. By combining theoretical analysis, field tests, and numerical simulation, the evolution laws of the fracture zone and water-conducting fractures were analyzed. The research results provide valuable technical references for coal mine projects under similar mining conditions^8^. The influence of the karst aquifer on overlying strata movement in a mining area was studied. Through numerical simulation, the failure modes of overlying strata under two conditions (with and without water-filled karst caves) were compared, and the maximum curvature and horizontal deformation values of overlying strata were obtained, which provided key parameters for the safe withdrawal of the working face^9^. With Youzhong Coal Mine as the research background, the evolution law of roof fractures under multi-seam mining conditions was explored by adopting a method combining theoretical analysis, numerical simulation, and on-site measurement. The study determined the maximum failure height of overlying strata and confirmed that under the condition of no structural influence, the No. 15 coal seam would not connect with the aquifer^10^.

Regarding the research on the evolution of overlying strata fractures, taking Yuhao Coal Industry as the experimental mine, based on the strata pressure and rock mass control theory, the spatiotemporal evolution characteristics of overlying strata fractures after coal seam mining were analyzed, and the fracture variation law of the separation space during the mining process was studied through numerical simulation. The research results are of great significance for mine gas prevention and control and water disaster prevention and control^11^. To study the influence of faults on the evolution law of mining-induced fractures in coal seam groups, taking Tucheng Coal Mine as the experimental mine, the evolution law of fractures during coal seam group mining was analyzed by using similar simulation and numerical simulation methods. The results show that: when the mining distance is the same, there are differences in the stress distribution characteristics of different coal seams; when the working face passes through the fault, the fracture evolution and development speed are extremely fast, the roof fragmentation is serious, and the fracture evolution shows irregularity^12^. Taking Xinhu Coal Mine as the experimental mine, the evolution characteristics of overlying strata fractures under fully mechanized top-coal caving (FMTC) mining conditions with a hard roof were studied. They applied the Brillouin scattering optical frequency domain scattering technology to the physical similar model test technology and successfully completed the research on the law of overlying strata fractures. The results show that this method has high effectiveness, and the research results are consistent with model analysis and theoretical analysis, as well as in line with the actual mine conditions, which can provide technical support for the research on fracture evolution laws under similar conditions^13^. Using microseismic detection technology, research was carried out on the evolution of roof fractures in the mining face of No. 1 Coal Mine of Pingyu Coal and Electricity Co., Ltd.Through testing, the failure locations of microfractures were accurately determined, and this monitoring system can comprehensively monitor the influence of roof mining on the aquifer during the working face withdrawal process, providing technical support for mine water disaster prevention and control and ensuring the safe mining of the working face^14^. Taking Nanling Coal Industry as the experimental mine, the deformation and instability problem of soft rock roadways was analyzed. Based on the stress-strain relationship of soft rock, they constructed a zoned failure model of roadway surrounding rock, and explored the fracture propagation law and stress distribution characteristics of the surrounding rock of soft rock roadways through numerical simulation. The research results provide technical support for the deformation and instability control of soft rock roadways^15^.

This paper takes a coal mine in Shanxi as the test mine, uses the staged water injection method, borehole television method, and microseismic method to detect the failure height of the overlying strata in the fully mechanized caving mining face, digitally analyzes the relationship between fracture dip angle, quantity, and width before and after mining, and conducts numerical simulation tests on the fracture evolution during the mining process^16^.

## Engineering overview and overburden engineering geological types

The coal mine has a production capacity of 5.0 Mt/a and mainly mines the No. 3 coal seam, which is located at the lower part of the Shanxi Formation. The coal seam has a simple structure and is nearly horizontal. The 3305 working face is selected as the test face, with an average coal seam thickness of 6.62 m, an average working face burial depth of 461 m, a dip length of 243 m, and a strike length of 1301.5 m. The immediate roof of the working face is sandy mudstone with a thickness of approximately 2.9 m, and the basic roof is fine-grained sandstone with a thickness of approximately 11.8 m. The average original gas content in the coal seam is 6.24 m³/t. The fully mechanized top-coal caving mining method is adopted.

According to physical properties, lithology, primary structure, and other characteristics, the No. 3 coal seam and overburden are divided into five engineering geological types:

1. No. 3 coal seam: It is distributed in a banded pattern with micro-fractures inside, and has low compressive strength. The coal seam outcrop is abnormally fragmented due to weathering.
2. Fine sandstone and sandy mudstone type: Mainly composed of fine-grained sandstone and sandy mudstone, with quartz and feldspar as the main components. The core in this type is intact and has high compressive strength, reaching up to 61.8 MPa.
3. Medium and coarse-grained sandstone type: Primarily composed of medium-grained and coarse-grained sandstone, with quartz and feldspar as the main particles. The core in this type is relatively intact, with moderate compressive strength, reaching a maximum of 57.9 MPa.
4. Coarse-grained sandstone type: Mainly composed of coarse-grained sandstone, with quartz and feldspar as the main particles. The core in this type has poor integrity and low compressive strength, with a maximum of 48.8 MPa.
5. Gravel and soil type: Mainly composed of gravel and soil. Due to severe weathering, complete cores cannot be obtained in this type.

## Analysis of failure characteristics of mining-induced overburden

### Determination of overburden failure feight by stage water injection method

#### Borehole layout

**Fig. 1:**
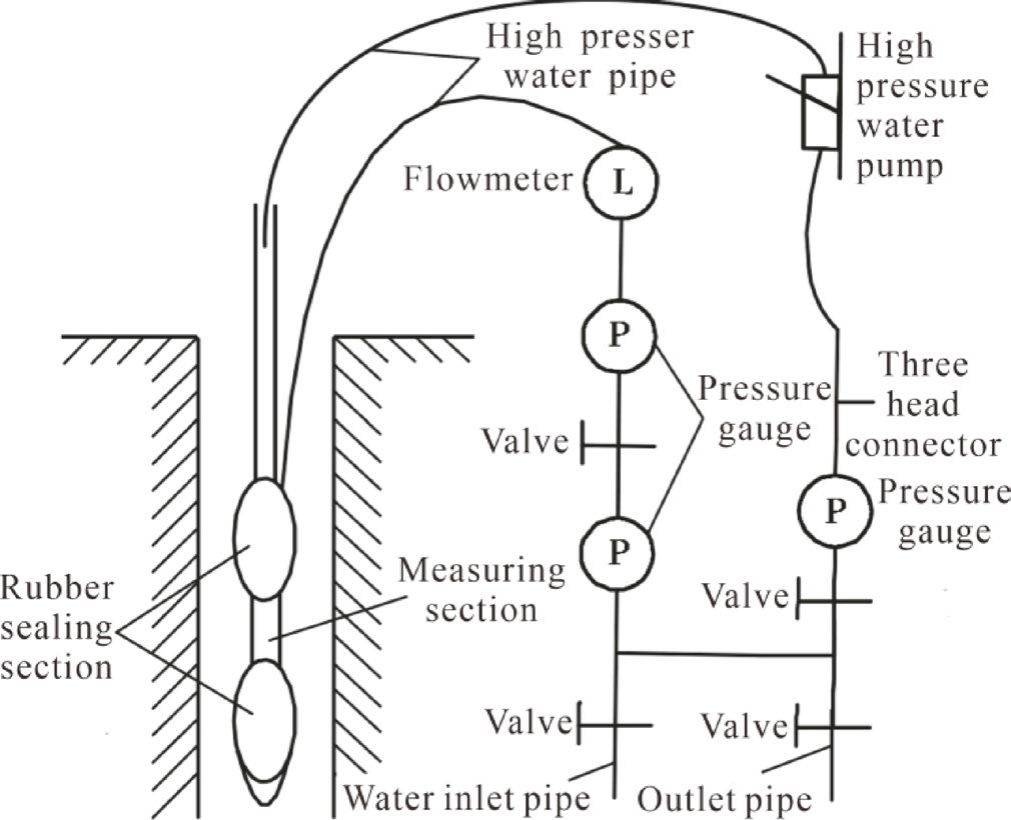
Structural diagram of stage water injection experimental equipment.

**Fig. 2:**
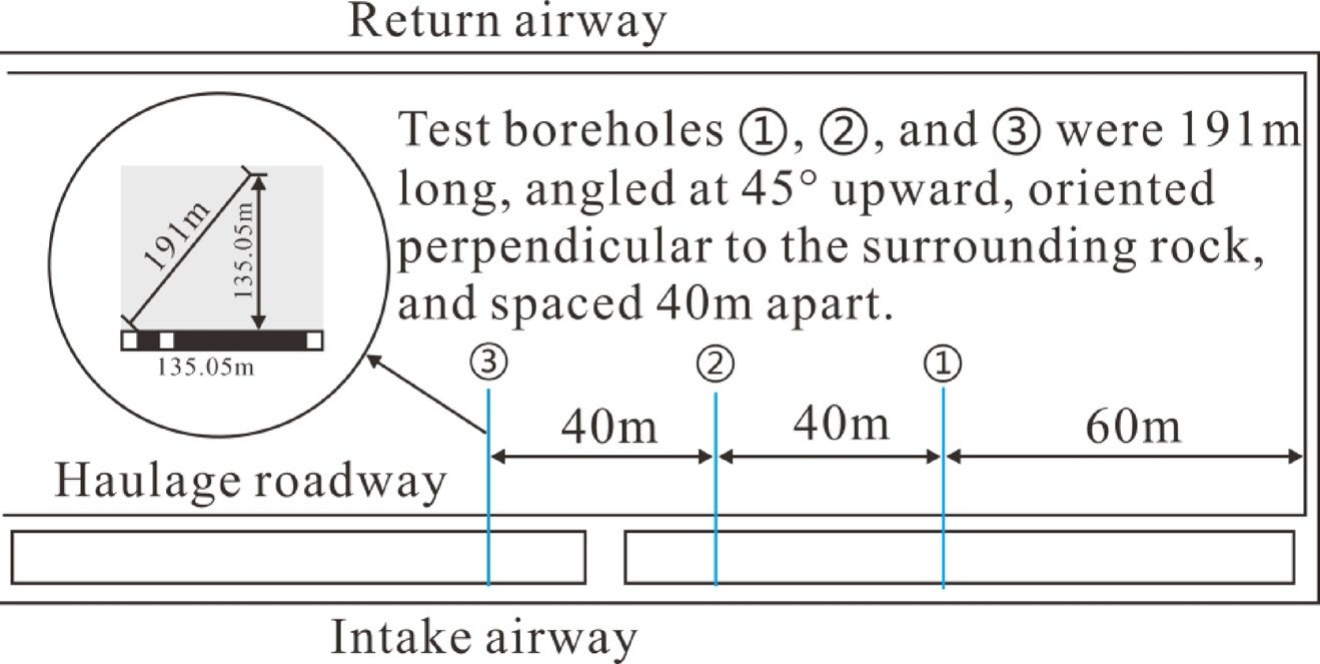
Sketch of boreholes layout.

To better understand the overburden failure height, the stage water injection method is used to determine it. The principle of the stage water injection method is that after mining^17^, the overburden undergoes different degrees of failure from bottom to top due to mining-induced effects, with varying degrees of fracture development. The overburden failure height is determined based on the difference in water injection volume at different stages^18^, and its principle is shown in Fig. 1. The experimental test section has a length of 1 m and a water pressure of 0.5 MPa. Three observation boreholes are arranged in the working face air intake roadway^4^, with an inclined borehole length of 191 m and an elevation angle of 45°. The borehole layout is shown in Fig. 2.

#### Test results and analysis

**Fig. 3:**
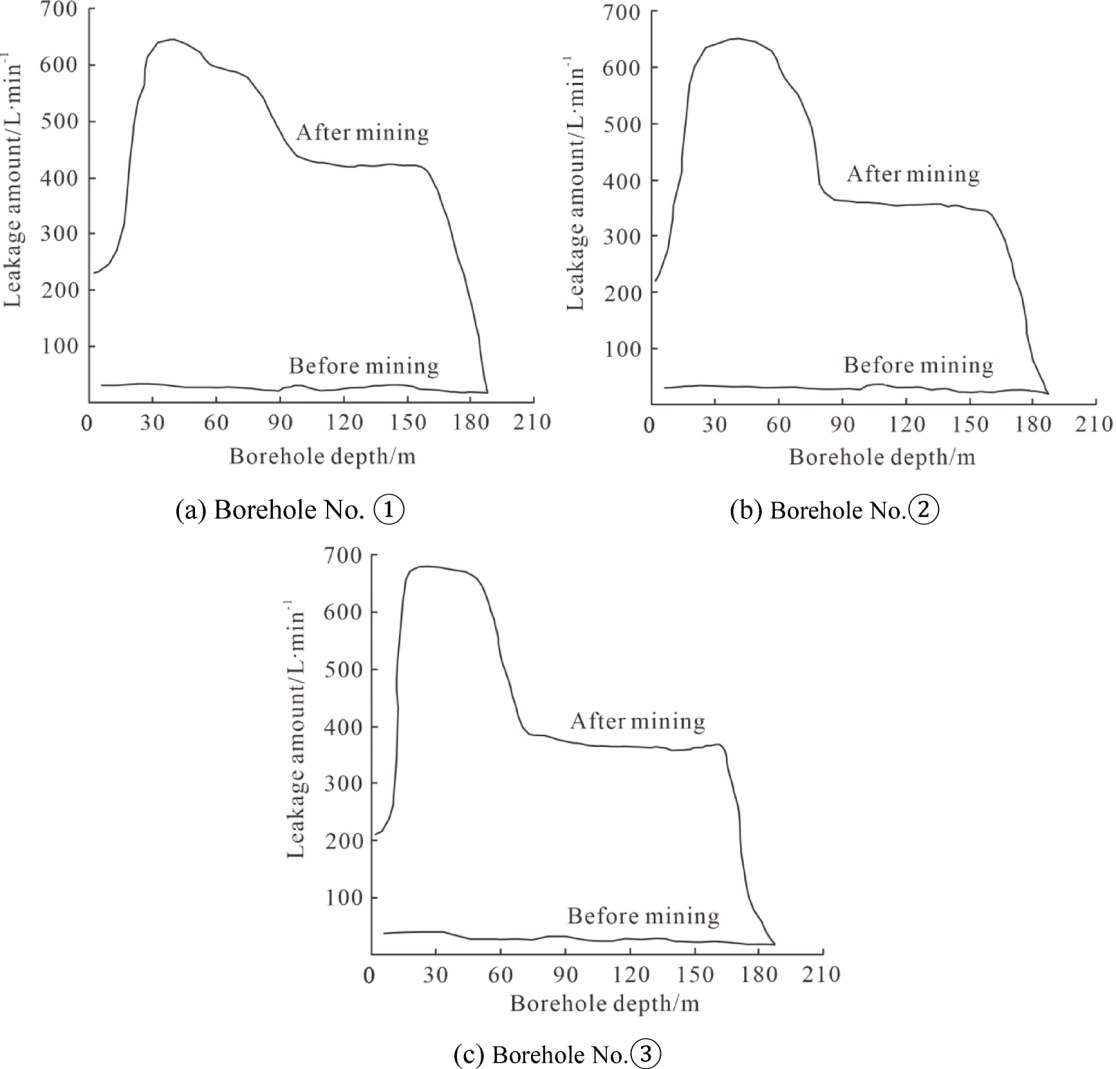
Stage water injection experimental data diagram.

The test results of the stage water injection experiment for the three boreholes are shown in Fig. 3.

As indicated by Fig. 3, the water injection volume in the boreholes was relatively low before mining, basically showing a linear distribution, with the water injection volume below 30 L·min⁻¹. This indicates that there were few primary fractures in the overburden before mining^19^, and the fracture development degree was low. After mining, the water injection volume curve of the boreholes showed a “step” type distribution, and the water injection volume recovered to the pre-mining level at a borehole depth of approximately 180 m. The water injection volume in the first step was 620–680 L·min^− 1^, which was 20.6–22.6 times that before mining^20^. The water injection volume in the second step was 360–420 L·min^− 1^, which was 12–14 times that before mining. According to the analysis of the fracture development characteristics of the “three zones” (caving zone, fracture zone, and bending zone) in the overburden after mining combined with the borehole water injection volume, the first step corresponds to the caving zone^21^, and the second step corresponds to the fracture zone. It can be seen that the caving zone heights of the three boreholes are 57.9 m, 55.1 m, and 53.7 m, respectively, and the development heights of the water-conducting fracture zones are 131.5 m, 132.2 m, and 131.5 m, respectively. Comprehensively, the development height of the caving zone is 53.7–57.9 m, and the development height of the fracture zone is 73.6–78.5 m.

### Borehole television observation of overburden fracture development and failure height

**Fig. 4:**
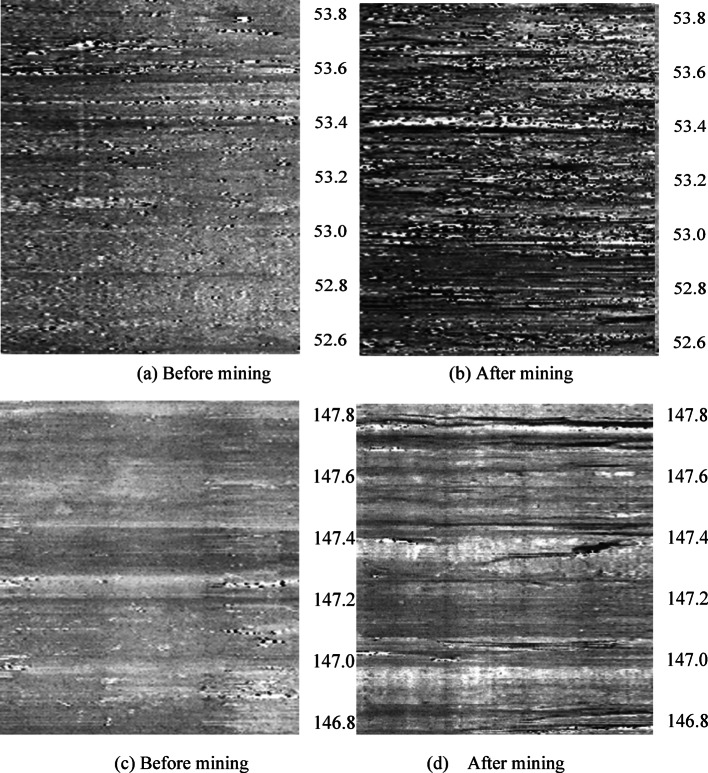
Fracture characteristic of boreholes after mining.

The borehole television is used to detect the development of overburden fractures, which can present the fracture development characteristics in the form of unfolded images^22^. Through the images, the fracture development characteristics and the boundary points of the “three zones” (caving zone, fracture zone, and bending zone) can be visually observed^23,24^. The borehole television detection data diagrams before and after mining for Borehole ① (52.6–53.8 m) and Borehole ② (146.8–147.8 m) are intercepted, as shown in Fig. 4.

As shown in Fig. 4, before mining [(a) and (c)], the number of overburden fractures was minimal, almost non-existent, indicating low fracture development in the overburden. After mining [(b) and (d)], the number of overburden fractures increased significantly, with higher fracture development.

Comparing Fig. 4a,b with Fig. 3a, it can be seen that the water injection volume in the borehole at this position was low before mining, but increased significantly after mining. Meanwhile, borehole television detection showed almost no fractures at this location before mining, while the number of fractures increased significantly after mining, which corroborates each other.

Comparing Fig. 4b,d, the number of fractures at position (b) after mining is higher than that at position (d), with higher fracture development, which is consistent with the zonal distribution characteristics of overburden fractures after mining.

**Fig. 5:**
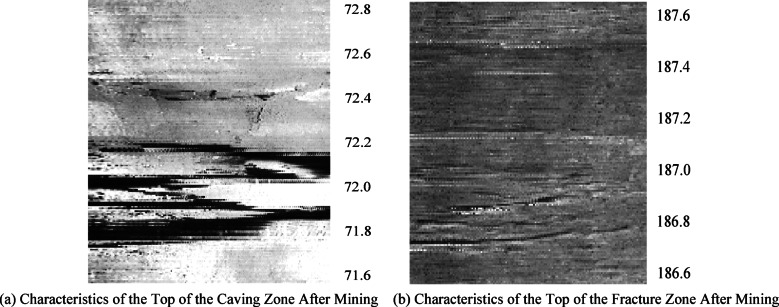
Characteristic of caving zone top and crack zone top.

According to the distribution characteristics of the “three zones” (caving zone, fracture zone, and bending zone) in the overburden after coal seam mining, the top height of the caving zone and the top height of the fracture zone were determined, and their data diagrams are shown in Fig. 5.

As shown in Fig. 5a, the top of the caving zone can be clearly observed. Based on the data from the other two boreholes, the development height of the caving zone is determined to be 51.1–52.8 m.

In Fig. 5b, there are almost no fractures above 186.9 m, which is similar to the fracture characteristics of the borehole before mining. Therefore, this position can be identified as the top of the fracture zone. Meanwhile, according to the conditions of the other two boreholes, the development height of the fracture zone is concluded to be 77.4–81 m.

### Detection of overburden failure height by microseismic technology

**Fig. 6:**
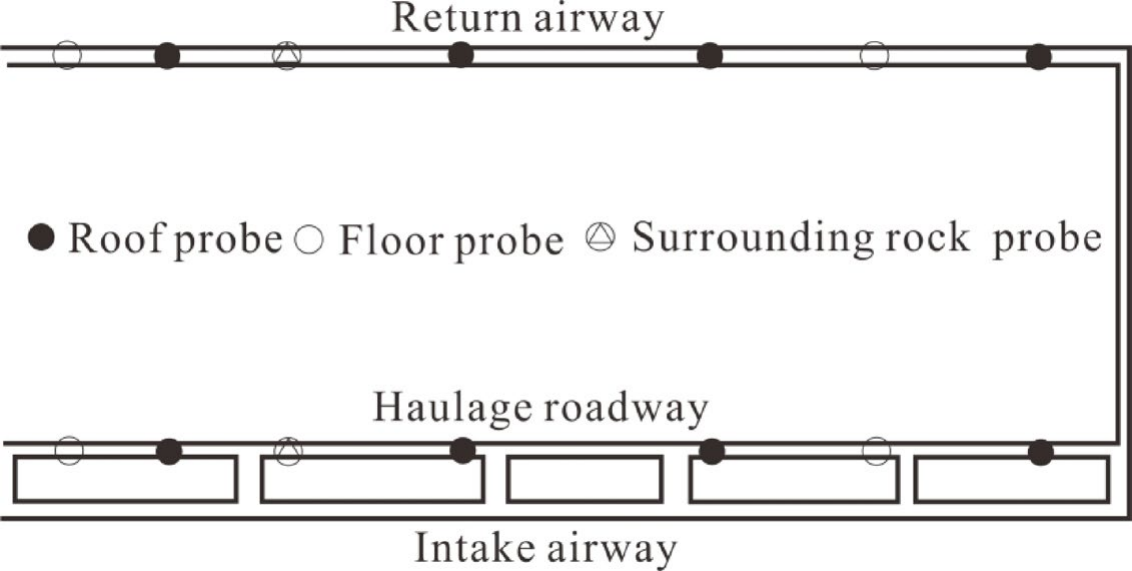
Sketch of microseismic monitoring.

Microseismic technology can conduct four-dimensional monitoring of overburden failure after mining, analyzing the failure characteristics of overburden based on events, energy, etc. The ARAMIS M/E microseismic system is adopted, with 10 sensors arranged within the working face range. The sampling rates are 50 and 100, the positioning accuracy is (± 50) m, and the probe layout is shown in Fig. 6.

**Fig. 7:**
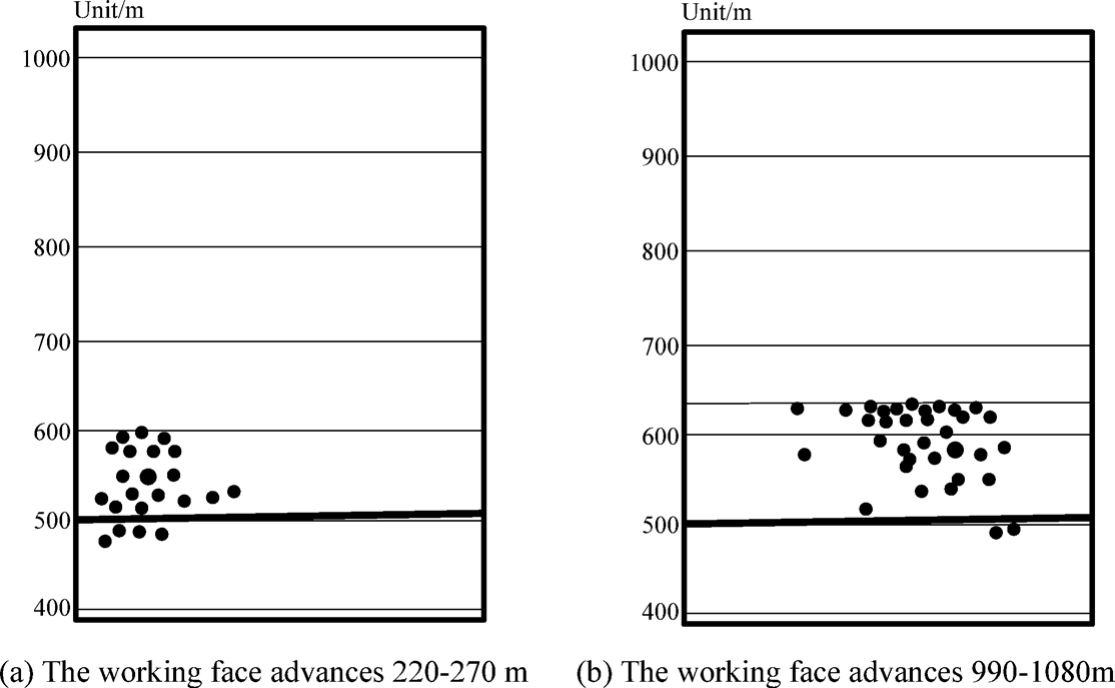
3305 microseismic information of overlying rock during working face mining.

Figure 7 shows the overburden microseismicity during the mining of the 3305 working face, with the energy points in the figure representing failure events with energy above 10,000 J.

As indicated by Fig. 7a, when the working face advanced 220–270 m, the overburden failure height was 101 m, and the maximum failure height was located in the upper-middle part of the sandstone.

Figure 7b shows that when the working face advanced 990–1080 m, the overburden failure height reached the boundary between sandstone and mudstone, ranging from 122 to 142 m. The lower sandstone was completely damaged, and the upper mudstone suffered partial damage.

Based on the overburden failure characteristics during the advancement of the 3305 working face, as the working face advanced, the goaf space gradually increased, and the overburden failure height gradually increased. However, when the overburden failure height reached the limit value, it no longer increased with the advancement of the working face. The maximum overburden failure height was 132 m.

## Digital analysis of mining-induced overburden fracture development

### Distribution characteristics of fracture dip angles before and after mining

**Fig. 8:**
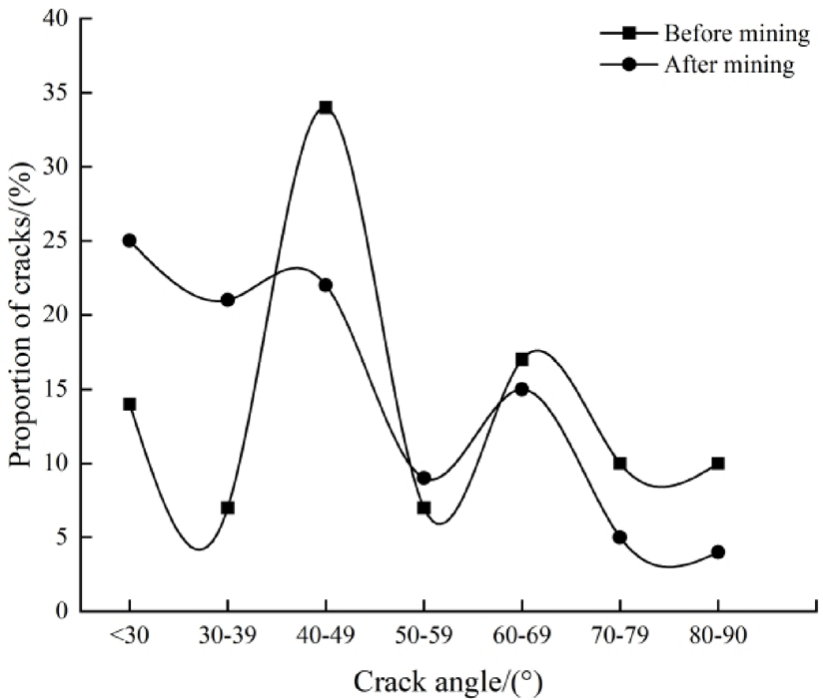
Dip angle distributions of crack before mining and after mining.

After vectorizing the borehole television imaging diagrams, it is concluded that Borehole ② detected 29 and 102 fractures before and after mining, respectively, with varying dip angles of fractures. Figure 8 shows the distribution of fracture dip angles before and after mining according to the dip angles of fractures. As can be seen from Fig. 8, among the 29 fractures before mining, fractures with a dip angle of less than 30° account for 14%, those with a dip angle of 30°–39° account for 7%, those with a dip angle of 40°–49° account for 34%, those with a dip angle of 50°–59° account for 7%, those with a dip angle of 60°–69° account for 17%, those with a dip angle of 70°–79° account for 10%, and those with a dip angle of 80°–90° account for 10%. It can be inferred that high-angle fractures are dominant before mining. Among the 102 fractures after mining, fractures with a dip angle of less than 30° account for 25%, those with a dip angle of 30°–39° account for 21%, those with a dip angle of 40°–49° account for 22%, those with a dip angle of 50°–59° account for 9%, those with a dip angle of 60°–69° account for 15%, those with a dip angle of 70°–79° account for 5%, and those with a dip angle of 80°–90° account for 4%. It can be inferred that the increased fractures after mining are mainly small-angle ones.

### Relationship between fracture quantity and fracture width before and after mining

**Fig. 9:**
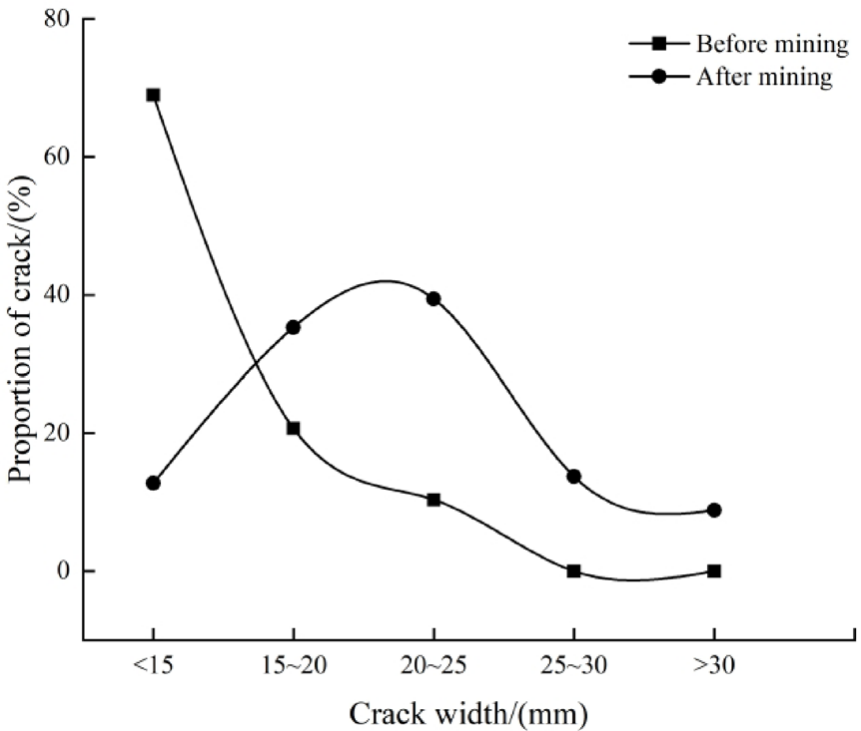
Distributions of crack width before and after mining.

**Fig. 10:**
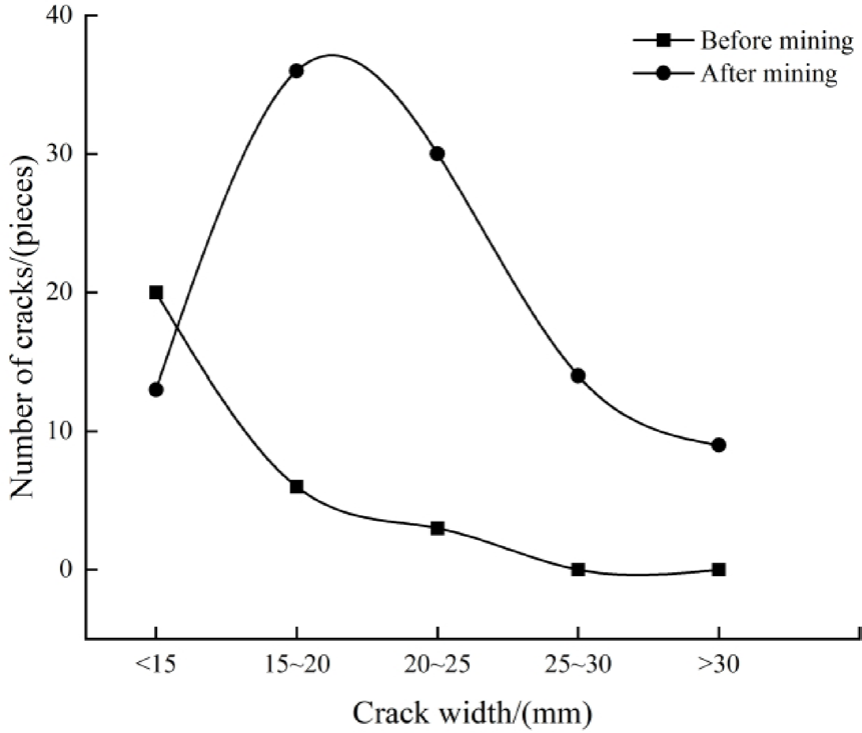
Relationship curves between width and quantity of cracks before and after mining.

Fracture width reflects the degree of fracture development. Under mining influence, fractures in the near-coal seam area are significantly wider. Statistical analysis of borehole fracture widths (as shown in Fig. 9) shows that before mining, fractures with widths less than 15 mm accounted for 68.97% of the total, while after mining, fractures with widths of 15–25 mm became dominant, accounting for 64.7% of the total. The curves of fracture width and fracture quantity before and after mining are shown in Fig. 10 through statistical analysis.

## Numerical test on overburden fracture development

### Test software

The UDEC numerical simulation software is used to simulate the failure characteristics of overburden during the advancement of the working face. To accurately simulate overburden failure, the strata near the coal seam are divided into dense blocks, while the rock blocks far from the coal seam are divided into sparse blocks to achieve fast calculation. The mechanical parameters are shown in Table 1.

**Table 1:** Physico-mechanical parameters of rock masses.

Lithology	Density/(kg/m^3^)	Compressive strength/MPa	Elastic modulus/MPa	Cohesion/MPa	Internal friction angle/(°)	Poisson’s ratio
Siltstone	2600	34.9	5.58	2.60	35	0.32
Medium sandstone	2410	57.9	5.52	2.40	33	0.30
Coarse sandstone	2290	48.8	6.27	2.20	31	0.31
Fine sandstone	2680	61.8	5.56	2.70	34	0.33
Coal	1420	11.1	1.38	1.10	19	0.34

### Simulation results

**Fig. 11:**
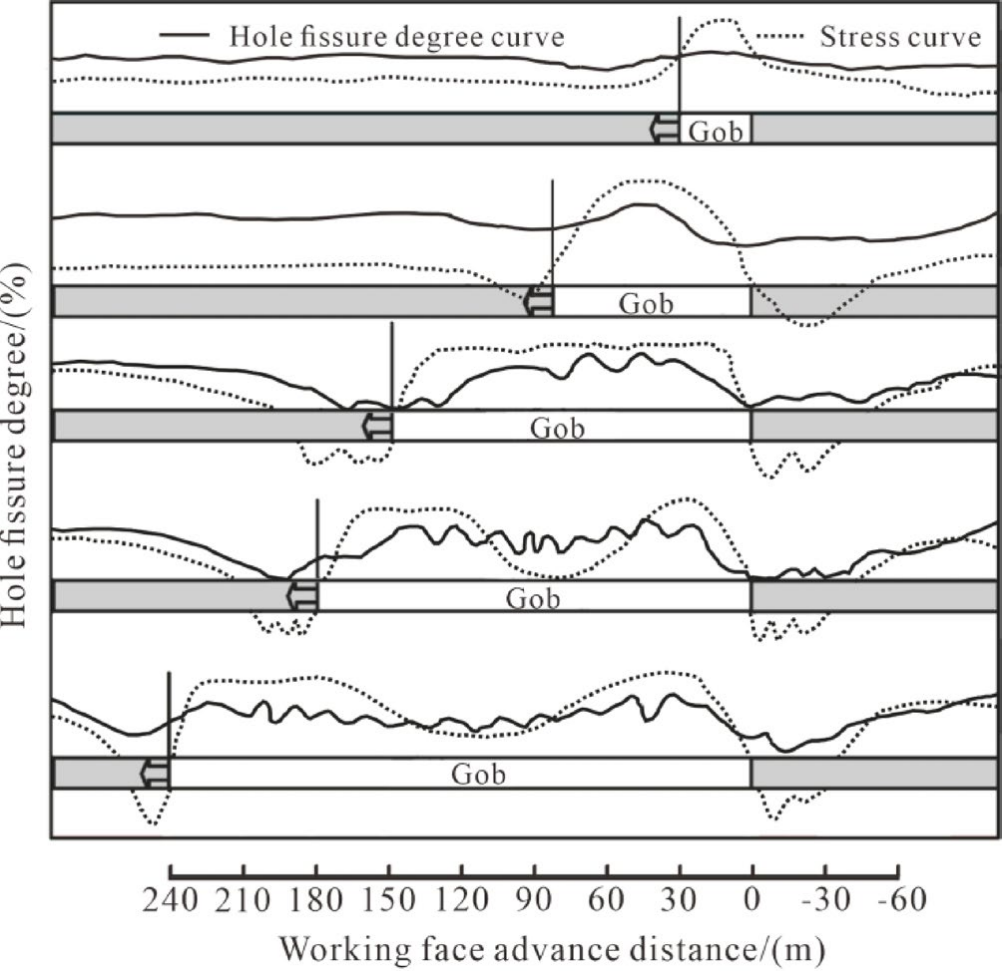
Distribution of porosity and stress and their evolutions with advancing different distances.

In this paper, the ratio of the comprehensive relative displacement difference between two points in the overlying coal-rock strata before and after mining to the pre-mining relative distance is introduced to represent the pore (void) fracture degree at that point, as shown in Fig. 11. The 3^#^ coal seam is excavated combined with the actual working conditions of the mine. This figure shows the fracture conditions in different sections of the goaf at different advancement distances, which is only a single observation; the oscillation degree of the curve is used to represent the contrast relationship between the pore (void) fracture degree and stress.

### Fracture characteristics of overburden strata during the test

1. When the working face advances 30 m, the immediate roof above the goaf collapses, and the pore-fracture degree inside the goaf is the highest at this time.
2. When the working face advances to 75 m, the lower sub-key stratum collapses while the main key stratum does not collapse, and the pore-fracture degree inside the goaf is the highest.
3. When the working face continues to advance to 150 m, the upper main and sub-key strata all collapse, the middle part of the goaf is compacted, and the porosity in the middle of the goaf begins to decrease.
4. When the working face advances to 180 m, the compaction degree inside the goaf further increases, and the increment of fracture degree in the middle of the goaf gradually decreases. However, due to the supporting effect of boundary suspended roofs and supports on the side of the working face coal wall and the side near the open-off cut, increment zones of fracture degree appear.

## Conclusions

1. Based on the observation and analysis results of the borehole stage water injection device, borehole television system, and microseismic technology, the overburden failure height of the fully mechanized caving face in this coal mine is determined to be 127.3–132.2 m.
2. Before mining, the fracture development degree was low. After mining, the number of fractures increased significantly, and the fracture development degree improved. Before mining, fractures were mainly high-angle and low-width. With the gradual advancement of the coal mining face, the number of fractures increased linearly, and the increased fractures were mainly low-angle and medium-width.
3. The numerical simulation results show that the main accumulation area of fractures during mining is around the coal wall, and the density distribution curve of overburden fractures is high at both ends and in the middle, showing a “wave” shape.

## Data Availability

The data supporting the findings of the present study are available from the corresponding author upon reasonable request.
